# Impact of Open Data Policies on Consent to Participate in Human Subjects Research: Discrepancies between Participant Action and Reported Concerns

**DOI:** 10.1371/journal.pone.0125208

**Published:** 2015-05-20

**Authors:** Jorden A. Cummings, Jessica M. Zagrodney, T. Eugene Day

**Affiliations:** 1 Department of Psychology, University of Saskatchewan, 9 Campus Drive, Saskatoon, Saskatchewan, Canada; 2 Office of Safety and Medical Operations, The Children's Hospital of Philadelphia, 3401 Civic Center Blvd., Philadelphia, Pennsylvania, United States of America; University of California, San Francisco, UNITED STATES

## Abstract

Research outlets are increasingly adopting open data policies as a requisite for publication, including studies with human subjects data. We investigated whether open data policies influence participants’ rate of consent by randomly assigning participants to view consent forms with and without discussion of open data policies. No participants declined to participate, regardless of condition, nor did rates of drop-out vs. completion vary between conditions. Furthermore, no significant change in potential consent rates was reported when participants were openly asked about the influence of open data policies on their likelihood of consent. However, follow-up analyses indicated possible poor attention to consent forms, consistent with previous research. Moreover, thematic analysis of participants’ considerations of open data policy indicated multiple considerations such as concerns regarding confidentiality, anonymity, data security, and study sensitivity. The impact of open data policies on participation raises complex issues at the intersection of ethics and scientific innovation. We conclude by encouraging researchers to consider participants as stakeholders in open data policy and by providing recommendations for open data policies in human subjects research.

## Introduction

When researchers adhere to open data policies, they freely provide their data for others to review, analyze, and publish. When adopted by academic journals, this policy recommends researchers provide data by uploading datasets to public repositories or providing them upon request. Indeed, the United States Office of Science and Technology Policy, in a February, 2013 memorandum, asserted that federally-funded research data should be made publicly available for access, search, and analysis [1]. Increasingly, publication in certain outlets, such as the Public Library of Science and Science, *requires* researchers to make their data open to public access.

The Open Knowledge Foundation (OKF) argues that open data promotes dissemination [2]. Open data policies are also argued to allow for transparency and accountability [3], as well as innovative uses of data. For example, open data for failed clinical trials might allow researchers to learn from previous null findings or avoid putting resources towards studies unlikely to lead to substantial findings [4]. Other reported advantages include maximizing data value, avoiding duplicate data collection and thus reducing research costs, lowering participant burden, allowing science to progress and be self-correcting, and promoting follow-up research [5]. At time of writing, The Panton Principles, [6] which outline principles for publishing open data, had 250 endorsers. Open data policies are currently subject to much debate within public health, medicine, and psychology. Editorials regarding open data policies have appeared in outlets that publish human subjects data such as the Journal of the American Medical Association [5] and the New England Journal of Medicine [7].

Critics of open data note several major concerns about these policies. These primarily relate to issues of intellectual property, researcher resources invested in data collection, and unwelcome competition for publications [5]. The re-use of clinical data for secondary analyses has been suggested to also present some concern, and questions have been raised regarding the rigor and relevance of such secondary data use, including a lack of novelty in secondary publications [8], as well as the statistical implications of previously collected data on hypothesis acceptance and rejection [9].

Debate regarding open data policies has focused primarily on the impact of open data policies on the scientific community itself. To date, this discussion has all but ignored those whose data is being openly shared: human participants. Given that each day 75 clinical (i.e., human) trials are published in biomedical journals [10], open data policies are poised to impact countless individuals who participate in human subjects research.

Open data policies allow research data to be utilized for purposes other than the original intention, without explicit permission [11]. This means participants’ data could be used for purposes other than those they originally consent for, by researchers with whom they did not consent, for a study they did not directly participate in. Furthermore, under open data policies research participants are unlikely to know when or for what purpose their data is being reused. We wondered if such open data policies might influence rates of participant consent. If informed consent is impacted by open data policy, this could have substantial implications for human subjects research. For example, if large numbers of potential participants declined to participate based on open data policies, samples will increasingly be biased or the usefulness of databases will be “diluted” [12].

### Current Study

Utilizing an experimental design, we randomly assigned participants to read a control consent form covering standard components of consent forms in psychological human subjects research (e.g., confidentiality, right to withdraw, risk associated with participating), or an experimental consent form that was identical except for inclusion of a description of open data policies. Participants were asked, at the end of the consent form, to click “consent” or “do not consent.” Participants were purposefully non-incentivized, in order to remove payment as a potential motivator. Following the consent forms, participants were presented with a recall task to assess their memory for the consent form components. Participants were then presented with a debriefing form outlining the purpose of the study and were openly asked about the potential impact of open data policies on their likelihood of consenting to participate in psychological research. That is, participants were provided with a Likert scale to report the impact of open data policies on their likelihood of consenting in research as well as an opportunity to provide an open-ended response explaining this rating. Thematic analysis, a method used to discern primary themes from qualitative data [13], was used to analyze these open-ended responses.

## Methods

### Participants

906 individuals accessed the Internet link for this study, which was hosted on Qualtrics and distributed via social media. Of these, 189 participants completed the entire study (i.e. “completers”) and 89 partially completed the study (i.e. “drop-outs”), an involvement rate similar to rates of “clicks” in other forms of public data collection such as marketing surveys [14]. On average, participants were 36.47 years old (*SD* = 10.06, *range* = 19–72); 74% of the sample was female (23% male, 3% not identifying a gender). There were no significant differences between completers and drop-outs on age or gender.

### Ethics Statement, Procedure, and Measures

This study was reviewed and approved by the University of Saskatchewan Behavioral Research Ethics Board. Further, all data is available from the corresponding author upon request. The online survey was administered via Qualtrics, a web-based survey software, with the link being distributed via Twitter. Upon visiting the study link, participants were randomly assigned to one of two consent form conditions. In the control condition, participants read a standard informed consent form for a study called “Interrelations Amongst Personality Variables,” which was selected to mimic a standard psychological study but also to maintain deception regarding the true purpose of this study. The consent form covered the standard domains of identifying the researchers, the study purpose and procedures, potential risks and benefits, confidentiality, participants’ right to withdraw, and how to contact the researchers with any questions or to obtain study results. In the experimental condition, participants read a consent form that was identical except for the description of open data policies. Specifically, under the confidentiality section of the consent form, it read “In addition, your data could be provided to a public data repository. That is, your research data may be required to be made publicly available in order to publish knowledge gained as a result of this study.” Participants were informed that this study would take approximately 10 minutes of their time, and were asked to click “consent” or “do not consent” upon reading the consent form.

Participants were then queried regarding six consent form components, some of which were presented in the consent forms (i.e., limits of confidentiality, withdrawal policy, and risks/benefits of participation) and some of which were not (i.e., funding source, potential publication outlets). The open data policy was only present in the experimental condition. Participants were asked to rate their confidence in recalling whether or not they had read each component, using a 5-point Likert scale ranging from 1 (*this was definitely not discussed*) to 5 (*this was definitely discussed*), and including an anchor value of 3 to indicate being unsure of whether that component was discussed. These questions were designed to form a manipulation check regarding the control vs. experimental conditions and to assess how closely participants paid attention to the consent forms.

Last, participants reviewed a debriefing form that outlined the purpose of the study. Following this study explanation, participants were asked to rate their likelihood of consenting in a research study with an open data policy, using a 7-point Likert scale ranging from 1 (*I would definitely not participate*) to 7 (*I would definitely be more likely to participate*), including an anchor point of 4 which indicated open data policies would not influence the participants’ decision. Participants were then asked in an open-ended response format why they had chosen that particular value.

We analyzed the open-ended responses using the thematic analysis model outlined by Braun and Clarke [13]. We chose to analyze and describe the entire data set in order to provide an overview of the decision points and constructs that might influence individuals’ interest in participating in studies with an open data policy. We used a semantic approach to data analysis, not attempting to extrapolate or infer participants’ underlying ideas or assumptions beyond what was stated in their written responses. In a few cases, this required us to eliminate responses because we could not understand the content or would have had to use inference in order to place that response within a theme. Last, we approached our data primarily from a realist epistemology. That is, we approached this from the perspective that researchers can theorize meaning from participant responses in a straightforward way, assuming a unidirectional relationship between meaning, experience, and language [13]. However, we also acknowledge that social context might impact participants’ responses. For example, many participants identified recent historical events related to data security and privacy that influenced their decisions regarding consent in an open data study.

## Results

### Quantitative Analyses

In order to test our hypothesis that open data policies would influence rate of consent, we intended to compare rates of consent vs. nonconsent in the experimental and control conditions. However, zero participants chose “do not consent.” We then compared rates of completion and drop-out within the two conditions, using study drop-out as a proxy for non-consent. No relationship was found between condition and study completion, χ^2^ (1, *N* = 278), = .706, *p* = .401. We then examined responses for our final survey item, which asked participants their likelihood of participating in a study with an open data policy: 24.1% of participants reported that they would be somewhat to definitely less likely to participate in a study adopting an open data policy, 49.2% reported that this policy would not affect their participation, and 25.6% of participants reported being somewhat to definitely more likely to participate in such a study. Thus, no significant change in participant consent rates was observable in our cohort.

### Manipulation Check

Our lack of differences in rates of consent between the control and experimental condition could alternatively be explained by participants’ lack of attention to consent forms. Three consent components (i.e., limits of confidentiality, data withdrawal policy, and risk/benefits of participation) were present in both conditions; two components (i.e., funding source and potential publication outlets) were present in neither condition; one component (i.e., open data policy) was present in the experimental condition only. If participants had read and noted the open data policy, we would expect significantly higher rates of confidence of recall on this manipulation check item in the experimental condition. However, there was no significant differences in scores for the control (*M* = 3.23) and experimental (*M* = 3.46) conditions; *t*(191) = -1.65, *p* = .101, with both group averages being close to 3, the anchor point for being unsure. Furthermore, 53.1% of participants in the experimental condition were unsure if they had seen an open data policy, when they had; 34.1% of participants in the control condition indicated that they had seen an open data policy on the consent form when they had not. Thus overall, results of our manipulation check indicate that participants’ recognition for consent form components was poor and that this lack of attention or recall might be an alternative explanation for our quantitative findings that open data policies do not impact rates of consent.

### Thematic Analysis

Whereas our quantitative analyses focused on examining whether or not open data policies impact rates of consent/non-consent in human subjects survey research, our thematic analysis focused on examining *why* participants might consent/not consent to participate in such research studies. In total, 89% of participants provided open-ended comments that were coded. This thematic analysis revealed four broad themes that influence participants’ decisions.

#### Theme 1: Trust vs. Mistrust of Confidentiality and/or Anonymity

Over half of participants indicated that considerations of trust related to confidentiality and anonymity of their data would influence their likelihood of consenting to participate in a study with an open data policy. Participants reported that *as long as* their data was kept confidential and/or anonymous, then an open data policy would be irrelevant to their participation in the study (e.g. “If the authors willingly made the data publicly available without revealing identities, than I would have no problem with it”).

Other participants reported a mistrust of confidentiality/anonymity. First, a subset of participants raised concerns that sharing of their open data would provide their data to 3^rd^ parties, who would not use their data responsibly, would not be responsible enough to provide confidentiality and/or anonymity, or would not care enough to keep data confidential (e.g., “…once it becomes public that exposes it to parties that might care more”). Participants also were concerned that 3^rd^ parties would use their data for profit. Second, some participants were concerned that their responses could identify them if the data points were triangulated (e.g., “If the subject matter of the study included deeply personal information, I would be concerned that even in aggregate and anonymized the data could be traced back”). Third, some participants expressed concerns that confidentiality/anonymity cannot be fully kept in an open data scenario. This could be due to data mining of open data, network traces, hacking, and concerns not yet identified.

#### Theme 2: Impact of Open Data Policies Depends Upon Study Sensitivity

A second major consideration for participants was the sensitivity of the particular study. These participants indicated that an open data policy would not affect their participation in studies that were not sensitive (e.g., “As long as the information didn’t have my name attached I don’t care if people can read it. I might change my answer if it was extremely personal information, but that’s it”).

#### Theme 3: Opinions About Relevance or Irrelevance of Open Data Policies

Responses included a variety of opinions about the relevance of open data policies to participation. Rather than reflecting the conditions under which participants would or would not consent, this theme reflected more straightforward and unconditional opinions on the relevance of open data policies for study participation. First, a subset of participants indicated that open data policies were irrelevant to their participation in open data studies. A second subset of participants clearly indicated that they would decline to participate in an open-data study (e.g., “I don’t want my information publicly available—ever”). Other participants were not willing to have their data automatically used for additional studies to which they did not individually consent (e.g., If I’m agreeing to participate in a study, I’m only agreeing to participate in one study… Not any and all future studies”).

#### Theme 4: Open Data Policies Promote Good Science

Almost one quarter of our participants reported beliefs that open data policies promote good science. For example, it was noted that open data policies promote transparency and honesty in research, that it can further and advance scientific knowledge, and lead to additional research (e.g. “I would maybe be more inclined because the data could potentially be used again and again”). In addition, participants noted that open data policies lead to studies that help other people (e.g. “Better for the public good”) and other researchers (e.g. “I’m a researcher myself, I want data to be available to help other researchers”). A number of participants also specifically noted that open data policies might promote efficient use of scientific resources.

## Discussion

Our quantitative results provided no evidence suggesting open data policies influence rates of consent: First, no participants chose to decline to consent. Second, there were no significant differences in rate of completion vs. rate of drop-out by condition (control vs. experimental). Third, 75.8% of participants reported being not impacted by open data policies or even more likely to participate, rates similar to those in the UK [12]. However, the results of our manipulation check, as discussed below, indicate that these results should be interpreted with caution and that quantitative designs might not be the best method for examining this issue.

Our analysis of open-ended responses revealed a much more complicated picture regarding the impact of open data policies on consent to participate in human subjects research and what factors might influence that consent. Many participants raised concerns related to mistrust of confidentiality and anonymity of data due to untrustworthy 3^rd^ parties, identification via triangulation of data points, hacking, or data mining. These concerns are not unfounded. For example, small combinations of demographic data can uniquely identify participants. In a study examining publicly available voter registration data, 87% of the American population could be identified based on zip code, gender, and date of birth [15]. Although one might argue that data points such as ZIP code might not be available, in many projects the location of the data collection is stated in manuscripts or can be inferred. This effect is particularly concerning when used as part of a *jigsaw effect*, where separate databases are cross-referenced in a way that can reveal participant information [16]. In sensitive studies participants might be easier to identify because they are the only participants within a database who possess certain characteristics (e.g., a rare medical or psychological condition) [16].

Participant concerns regarding data mining for profit also are founded. Hand [16] cites examples where the corporations AOL and Netflix both released large datasets of search queries and film ratings, respectively. Despite deidentifying both datasets, customers could be identified. Participant concerns related to distrust of 3^rd^ parties and data security are also founded. At time of writing, the Canadian National Research Council was forced to shut down computers to prevent hacking of sensitive information from cyberattacks [17]. Protected health information is protected in the United States by HIPAA precisely because it contains data that may be exploited to harm or defraud individuals. High profile events such as the release of classified documents to Wikileaks, or the domestic spying scandal involving the United States’ National Security Agency lend credence to concerns that personal data might not be kept secure.

Other participants noted concern with their data being used multiple times, reporting that they prefer instead to consider a single study and consent/not consent based upon the characteristics of that specific study, similar to previous results [18]. An additional subset of participants reported that open data policies were irrelevant to their considerations of consent. These participants reported feeling either compelled to help researchers conduct studies, or that they had a pro-Open Access ideology. Related to this, the final theme that emerged from our data indicated that some participants had positive views about participating in open data studies because of their belief that this promoted good science. These participants reported benefits that very closely map on to the advantages noted by the OKF.

Notably, our quantitative and qualitative analyses reveal a large discrepancy between participants’ actions and their self-reported concerns. We believe this highlights the complicated relationship between open data policies, participation, and informed consent. Further, it emphasizes the importance of utilizing multiple methods when studying this topic. Our results also highlight substantial concerns with informed consent practices, particularly those used for online surveys.

### Concerns Regarding Informed Consent

Results from our manipulation check raise significant concerns about participants’ potential lack of attention to online consent forms. First, the median amount of time completers spent on the *entire* study (4.30 minutes) seems insufficient for full comprehension of the material presented. Given this concern, we replicated this study with a small second sample (*n* = 38). This replication was identical except for the addition of a timer on the consent form itself. In this second sample, participants spent an average of 25.89 seconds (*SD* = 38.37, *range* = 2.95–212.45) reading the consent information, which is likely insufficient to read and comprehend our forms, which were 481 words (control condition) or 554 words (experimental condition) long. Online consent forms can tempt participants to simply “click past” them, with up to 35% of participants not reading or only skimming the consent form [19]. Our manipulation check questions further indicated poor recall. For example, 34% of participants in the control condition reported recalling reading an open data policy, which was not presented. Only 36.7% of participants who *did* see the open data policy recalled seeing it. In addition, 24.6% and 17.4% of participants, respectively, reported seeing two components that were not presented in either consent form (i.e., funding source, intended publication outlet).

Unfortunately, these results are not unique to our study. A substantial body of research indicates that participants might not always attend to or comprehend information presented in consent forms, even in “high stakes” or high-risk research. For example, participants have shown a lack of consideration or comprehension of consent forms in areas such as pediatric clinical drug trials [20], medical trials involving potential administration of placebo [21], rainforest conservation programs [22], research using MRIs [23] and genetics [24]. In some medical research studies participants might not even be aware they are participating in research [25]. Brody and colleagues [26] found that less than 20% of participants in psychological research viewed informed consent as an opportunity to decide about participation, despite this being the primary purpose of informed consent practices. Participants have reported not reading consent forms closely because of a perception that they are all the same [27].

Researchers who are separated from participants by location and time (e.g., in online studies) might understandably argue that the burden of consent falls on the participant. However, such consent does not protect researchers from future concerns. For example, Kramer et al [28] noted that their recently published study examining mood contagion via social media was consistent with Facebook’s Data Use Policy. However, this did not preclude concerns from scientists and participants regarding the potential lack of *informed* consent and ability for participants to withdraw from that particular study [29]. Current models of informed consent assume the following steps: autonomous individuals are given information about a study, provided with time to assess that information, and then make a conscious decision about whether to participate [21]. However, what is assumed versus what is achieved in practice might be different, just as consent and *meaningful* consent are also different. We recommend that researchers, regardless of their use of open data outlets, be attuned to issues related to informed consent and attention to consent forms.

### Limitations and Future Directions

Some limitations to this study should be mentioned. First, we utilized a small convenience sample, collected via social media and a snowball sampling method (i.e., potential participants sharing the study link with others). This specialized sample might not represent the larger population of potential research participants. Related, our consent forms described psychological research. While this might represent a large area of online survey research we cannot necessarily be sure if our results generalize to open data policies as applied to other disciplines of human subjects research. Last, participants provided relatively short open-ended comments for qualitative analysis. Future research on open data policies and informed consent should aim to collected broader samples, targeting multiple areas of human subjects research (e.g., biomedical, genetic, political, economic). Future studies should also included more detailed interviews with open data stakeholders, including both researchers and potential participants, which would allow for more in-depth qualitative analysis of beliefs related to open data and informed consent.

### Implications and Recommendations

Our findings have a number of implications for open data policies and human participants. First, researchers and the public (i.e., potential research participants) might have contrasting views about the importance of open data policies. Nearly all discussions of the advantages and disadvantages of open data policies seem to hold researchers and publication outlets as the primary stakeholders—ignoring the research participants whose personal data and experiences provide the entire basis for human subjects research. Our data indicate that many participants are not convinced of the advantages of open data. They raised several credible concerns regarding confidentiality and privacy. Furthermore, as open data repositories are online, open data and online data security are inextricably entangled issues. As Anderlik and Rothstein [17] note, assurances of confidentiality are somewhat meaningless without attention to data security. Participants have the right to be informed of open data policies. Thus, our first recommendation is that researchers and journal editors consider a two-stage consent process, whereby participants can consent/not consent to participate in a study and then *separately* consent/not consent to have their data uploaded to an open data repository. Although this approach has limitations—not all data points will be uploaded to the repository, and any alternate methods of consent might inevitably lower rates of participation [30]—it is potentially more respectful of participants’ wishes and consistent with consent as a process, not a discrete event [21]. Otherwise, if researchers submit data to an open data outlet, they might be operating against the wishes of a large subset of their participants. This seems ethically questionable.

Second, as journals and research organizations develop open data policies, we recommend that they include participants as stakeholders to consider. Potential participant views should be included when developing open data policy. Researchers seem to be facing a crisis of confidence regarding human data and our ability, or even our intentions, to be safe and responsible with that data. We are seen as competent, but not trustworthy, by the public [31]. Interestingly, concerns raised by potential research participants are similar to concerns the public has identified regarding corporations obfuscating their intentions regarding data [32], corporate data collection and data sharing [33], and uses of data for alternate purposes [34]. The general public might not understand the rights of research participants, or that researchers might be held to a higher ethical standard than private companies. We recommend that researchers continue to educate the public as much as possible, including specific and thorough conversations with participants during recruitment.

Third, we recommend explicit open data exemptions for sensitive participant data in order to protect confidentiality and anonymity. This is imperative for participants with rare or identifiable conditions. Such exemptions for sensitive data exist in areas such as ecology and evolutionary biology [35]. The requirement of open data for publication should include an assessment of risk to participants, and significant risk of reidentification or harm should exempt researchers from open data reporting requirements.

Open data policy as applied to human subjects research represents an area with few regulations. What data should be uploaded? How may it be accessed—and who can access it? For what purposes can open data be utilized? Are there “wrong” ways to use open data and, if so, who will police this? The journal outlet that publishes a specific study currently sets open data policies; there are no uniform policies. Other researchers have recommended the creation of review panels to determine access to open data. Such panels have been implemented by drug companies, including GlaxoSmithKline [4] and can be requested, for example, from www.clinicalstudydatarequest.com. Others have recommended fee for service access to open data as a mechanism for limiting distribution of participants’ data [36]. Overall, open data repositories need reasonable-use policies that allow properly-consented open data to be accessed and used for legitimate research, but not to be web crawled by any party.

In general, current ethical practices might not automatically meet the needs of open data policies and their related concerns regarding online data storage. As Berry [37] notes, “the Internet is in a constant state of flux and technical change… ethical considerations and responses will need to adapt accordingly” (p. 324). Unfortunately, ethical standards are often adjusted in response to crises and failures of ethics codes to meet research challenges, rather than being proactively designed [21]. Open data policies are poised to make a substantial impact on the efficiency and potential innovation of human subjects research. Our final recommendation is that human subjects researchers, as a whole, need to continue to discuss and anticipate the ethical implications of open data and create policy guidelines that are proactive, rather than waiting to respond reactively to the potential open data failures of the future. At the individual researcher level, researchers need to thoroughly consider the ethical implications of open data for their own data collection and dissemination, as ultimately they are the guardians of the data their participants provide.
