# Correction: Impact of Open Data Policies on Consent to Participate in Human Subjects Research: Discrepancies between Participant Action and Reported Concerns

**DOI:** 10.1371/journal.pone.0131852

**Published:** 2015-06-25

**Authors:** Jorden A. Cummings, Jessica M. Zagrodney, T. Eugene Day

The following information is missing from the Funding section: This work was supported by a Saskatchewan Health Research Foundation Establishment Grant awarded to Jorden A. Cummings, Ph.D.
